# Analysis of 16S rRNA environmental sequences using MEGAN

**DOI:** 10.1186/1471-2164-12-S3-S17

**Published:** 2011-11-30

**Authors:** Suparna Mitra, Mario Stärk, Daniel H Huson

**Affiliations:** 1 Center for Bioinformatics ZBIT, Tübingen University, Sand 14, 72076 Tübingen, Germany

## Abstract

**Background:**

Metagenomics is a rapidly growing field of research aimed at studying assemblages of uncultured organisms using various sequencing technologies, with the hope of understanding the true diversity of microbes, their functions, cooperation and evolution. There are two main approaches to metagenomics: amplicon sequencing, which involves PCR-targeted sequencing of a specific locus, often 16S rRNA, and random shotgun sequencing. Several tools or packages have been developed for analyzing communities using 16S rRNA sequences. Similarly, a number of tools exist for analyzing randomly sequenced DNA reads.

**Results:**

We describe an extension of the metagenome analysis tool MEGAN, which allows one to analyze 16S sequences. For the analysis all 16S sequences are blasted against the SILVA database. The result output is imported into MEGAN, using a synonym file that maps the SILVA accession numbers onto the NCBI taxonomy.

**Conclusions:**

Environmental samples are often studied using both targeted 16S rRNA sequencing and random shotgun sequencing. Hence tools are needed that allow one to analyze both types of data together, and one such tool is MEGAN. The ideas presented in this paper are implemented in MEGAN 4, which is available from: http://www-ab.informatik.uni-tuebingen.de/software/megan.

## Background

Metagenomics is the study of the genomic content of a assemblage of organisms, obtained from a common habitat or an environmental sample of microbes. With the progress in the throughput and cost-efficiency of sequencing technology, there is a rapid increase in the number and scope of metagenomic projects. Two possible ways to analyze the taxonomic content of an environmental sample are either to perform a random shotgun sequencing of the DNA of the sample, or to use a targeted approach in which only one particular gene is amplified and sequenced. The latter is sometimes called *amplicon sequencing.*

As rRNA gene sequences are present in all living cells, these sequences (16S or 18S rRNA) are widely used for phylogenetic studies and also as the target of amplicon sequencing [1,2]. There are a number of tools for the analysis and comparison of 16S or 18S data, such as DOTUR [3], MOTHUR [4], SINA aligner at the SILVA website [5], RDP [6] and EstimateS [7]. More recent tools include MLtreemap [8], UniFrac [9] and pplacer [10] and QIIME [11].

MEGAN (“MEtaGenome ANalyzer”) [12] is widely used to perform the taxonomic and functional analysis of large metagenomic datasets. Previous versions of MEGAN could only be applied to random shotgun sequences. One of the new features released with the version 4 of MEGAN [13,14] is the ability to analyze 16S sequences. The aim of this paper is to describe this new approach in more detail.

We will illustrate how to apply MEGAN4 to rRNA sequences using an example dataset of ≈ 4000 published 16S sequences from [15] (obtained from a set of mice children and referred here as ‘mice-data’). The ideas presented in this paper are quite simple. The main merit of this work lies in the integrated implementation of the methods in the form of a user-friendly program, which can be used by biologists to analyze their 16S datasets in the context of other types of datasets.

## Methods

The aim of this work is to support the analysis of the result of a BLAST comparison of 16S rRNA against the SILVA database. SILVA result files do not contain the information on the species and/or the strain from which a reference sequence was obtained. Hence, we created a mapping file that maps SILVA accession numbers to corresponding NCBI taxon IDs. This mapping file is used by the “accession lookup” feature of MEGAN4 to identify the related species. An advantage of this approach is that no modification to the original SILVA database are required and it is possible to include additional information on the species/strain name when creating the mapping file. Moreover the mapping file is very small and can be updated with ease.

### Data extraction from the SILVA-ARB file and the NCBI file

**Figure 1:**
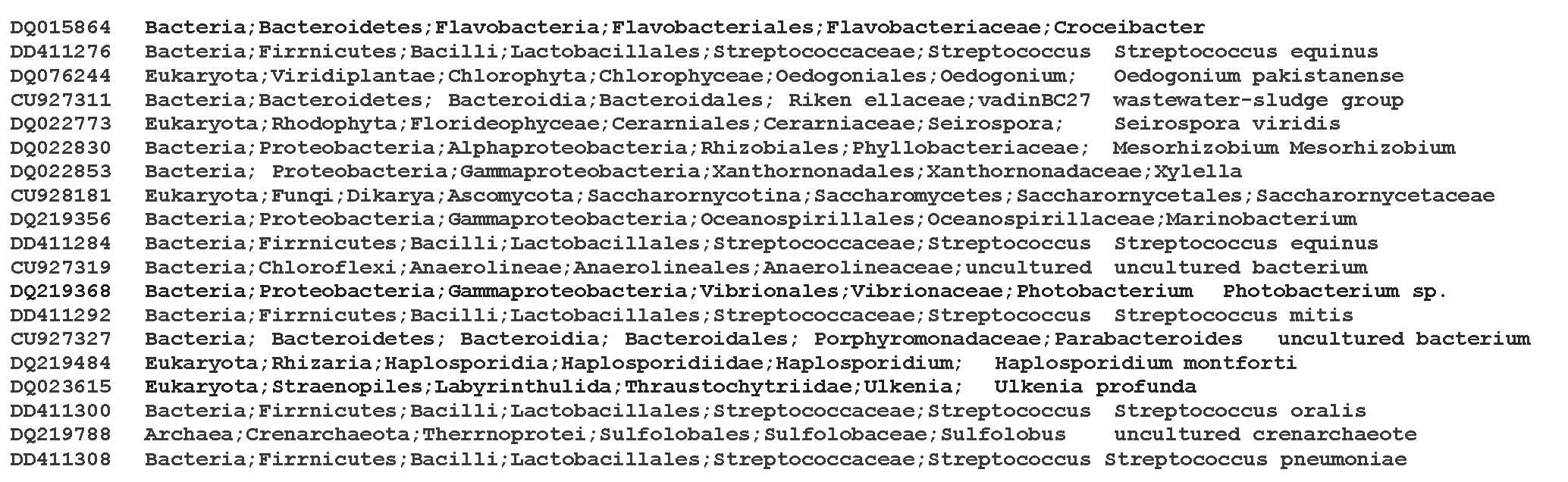
A part of the final SILVA file obtained using ARB software from SILVA website. The entries are exported and used in the mapping file.

To create the mapping file some information on the SILVA sequences such as accession numbers, the corresponding full taxonomic path and species/strains information are needed. A file containing these information can be created by exporting the SILVA database using the ARB-software (Available at: [16]). The entries are exported using the “NDS field export” function. A part of the final data file (referred to as the SILVA file) is presented in Figure 1.

**Figure 2:**
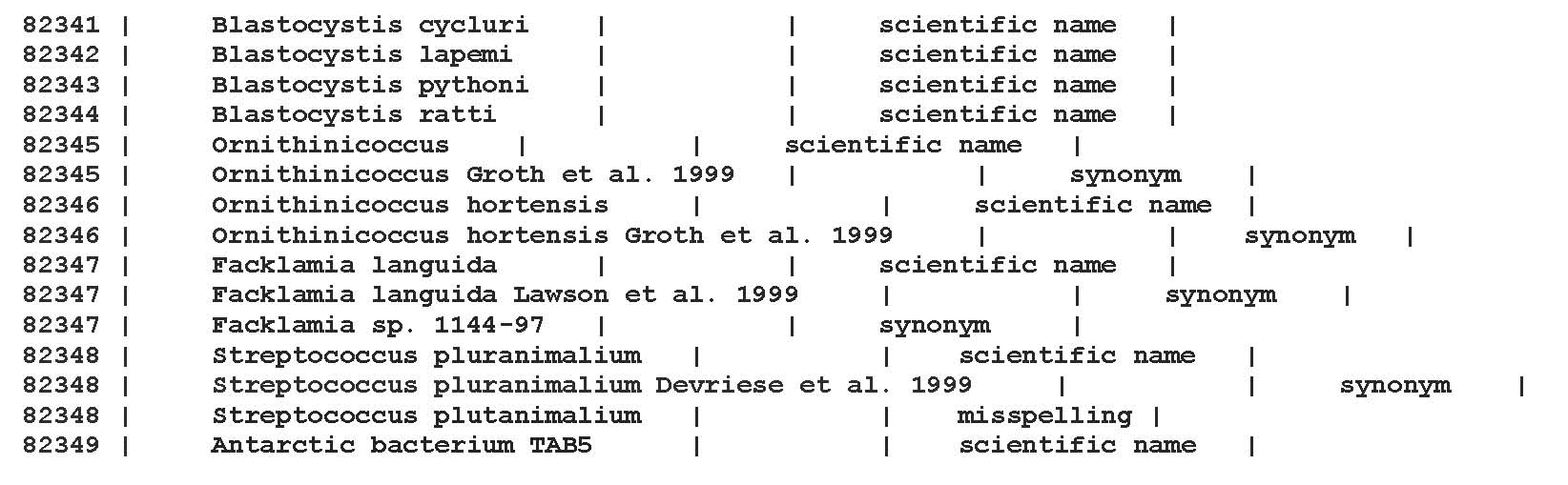
A part of the NCBI taxonomy file downloaded from the NCBI archive. This file is used to obtain a mapping between NCBI taxon IDs and the associated NCBI taxon names.

In addition, the algorithm also requires the input of a file containing a mapping between NCBI taxon IDs and the associated NCBI taxon names. For this purpose we downloaded the ‘names.dmp’ file (referred to as the NCBI taxonomy file) contained in the ‘taxdmp.zip’ archive from [17]. Beside the scientific name this file includes synonyms, equivalent names and misspellings. Those additional notations provide a higher chance for successful name matching. A part of this file is shown in Figure 2.

### Mapping algorithm

Our simple mapping algorithm starts by computing a hash map between all NCBi taxon names, synonyms, equivalents names and missing spellings, on the one hand, and all corresponding taxon IDs, on the other. Then each entry in the SILVA file is compared against the NCBI map. If a match to a taxon name is found, then the Silva entry is mapped to that taxon, unless the taxon name contains one of the keywords ‘uncultured’, ‘unidentified’ or ‘metagenome’, in which case the lowest taxon entry from the SILVA full taxa description is taken. If neither case is successful, then we change the capitalization of the Silva entry and retry the matching step.

The algorithm is illustrated by the examples in Table 1. When a species name is supplied, as in the first example, the algorithm tries to find this name in the NCBI map and then writes the corresponding NCBI ID for this name and the Accession ID of the read to the mapping file. In this example, a match was found. The Accession *AB365303* is assigned to the NCBI ID *336503*, which represents *Homalopoma granuliferum*, and is written to the mapping file.

**Table 1:** Examples taken from the SILVA file for illustrating the mapping algorithm (taxonomic paths shortened when necessary).

Accession	Taxonomic path	Full name
AB365303	Eukaryota; Metazoa;[…]Turbinidae; Homalopoma	Homalopoma granuliferum
AY548990	Bacteria; Firmicutes;[…]Family XII Incertae Sedis; Fusibacter	uncultured bacterium
AY763126	Eukaryota; Metazoa; Nematoda; environmental samples	uncultured nematode

As a second example (Table 1), the keyword ‘uncultured’ appears in the species name. In such a situation the taxonomic path is used to assigning this read to a taxa. To be precise, the lowest taxonomic entry which is in this case *Fusibacter* is considered. This name is found in the NCBI hash map and the read assigned to the ID *76008*. If there was no hit for *Fusibacter*, the next higher taxonomic entry would be used for searching (in this case: *Family XII Incertae Sedis*). If this search also failed to find a hit, then this procedure is repeated until the highest taxonomic entry for this read (here: *Bacteria*) is reached and a hit is to be expected.

The last case illustrates an example of combining two unwanted keywords (Table 1). In the species name the keyword ‘uncultured’ again appears. The lowest taxonomic entry is also rejected by the *searchFullTaxa*-method because the keyword ‘environmental samples’ is detected. So this read is finally assigned to NCBI ID for *Nematoda*.

### Test dataset

In order to test the analysis method with the created mapping file first we used published 16S sequences from [15] (≈ 4000 reads obtained from mouse guts (all mice children data) referred to here as ‘mice-data’).

First the ‘mice-data’ is aligned against the SILVA ribosomal RNA sequence database [5] using a variant (BLASTN) of the program BLAST [18]. Furthermore, we aligned the dataset against NCBI-NR database (of non-redundant protein sequences [19]) using BLASTX, expecting to see no hits as the NR database is not supposed to contain any 16S sequences, as it is a database of protein sequences. For both the cases for aligning the sequences using BLAST we used a very relaxed threshold in order to allow almost all the mappings. But while importing it in MEGAN we used a threshold of *Min Score*=*120*, *Top Percent*=*10* and *Min Support*=*5*, which enables a conservative assignment.

### Importing datasets in MEGAN 4 using the mapping file

**Figure 3:**
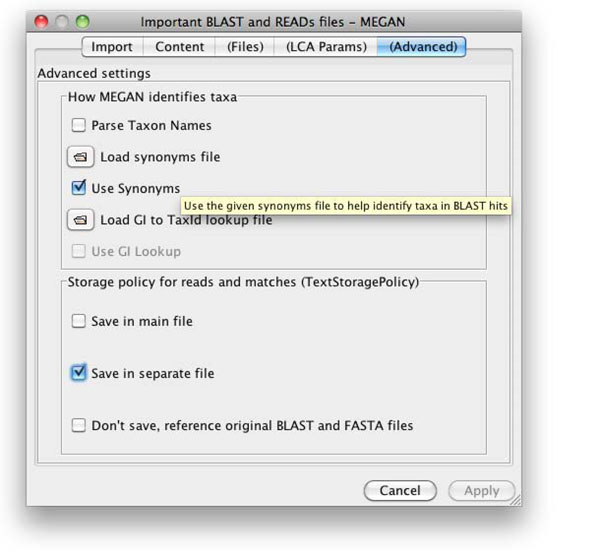
Advanced-tab in the *Import from BLAST*-menu for BLAST output files requiring the mapping file.

When importing BLAST output files produced by comparing against SILVA database some adjustments need to be made in comparison to the case of regular BLAST files compared against NCBI. After selecting the BLAST output file in the *Import from BLAST*-menu item the option *Use Synonyms* needs to be enabled in the *Advanced*-tab, providing the previously described mapping file (as shown in Figure 3).

This tells MEGAN4 to use the mapping file to assign the accession number of a hit in the BLAST output file to a taxa, before trying to make taxon names. Before opening “regular” BLAST output files these changes must be revoked.

### Comparison with other services

To compare the performance of the MEGAN4 analysis based on a BLASTN comparison of the reads against the SILVA database, we applied a number of different analysis tools to the mice-data. In more detail, we ran the data through the RDP web server [6] (using ‘Confidence threshold’: 80%) and the SINA aligner at the SILVA website (using default settings) [5], and a Greengene-, RDP- and SILVA-based analysis offered by MG-RAST web service [20] (all analyses are as of July 12, 2011). For MG-RAST analyses the e-value cutoff for sequences matches to these annotation sources (Greengene/RDP/SILVA) was set to 1 × 10^4^ with a *Min. % Identity Cutoff* as 90%. We didn’t specify *minimum alignment length* in order to allow all the assignments with previous threshold. We extracted our result at genus level in order to compare the analyses in depth. As MG-RAST does not produce a result in a hierarchical structure we certainly loose many hits that couldn’t attain the threshold at ‘genus level’. For comparison purpose we put those reads that are not available at ‘genus level’ analysis as ‘No hits’.

MEGAN4 is able to directly import the results obtain from the RDP website and also the results obtained from the SILVA website. For importing the the SILVA result users need to select *Import from BLAST*-menu item using the option *Use Synonyms* as mentioned above. For RDP analysis results users need to download the resulting text file from the “Classifier:: Assignment detail” page. For importing the analysis directly from SILVA website, users need to download the “log file” after running the website’s aligner. MEGAN 4 is then able to read both the files using the standard ‘Import from BLAST’ dialog. MG-RAST results can be saved and imported using an importer for CSV (comma separated value) files (using only two columns ‘genus’ and ‘abundance of reads’ without any header).

## Results

### MEGAN 4’s SILVA based analysis

**Figure 4:**
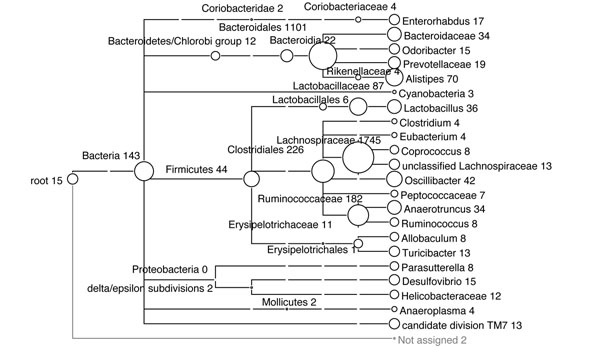
MEGAN4 analysis result for ‘mice-data’ against the SILVA database. Numbers in taxonomic tree indicate the number of assigned reads to each taxon collapsed at ‘genus’ level of NCBI taxonomy.

The results produced by MEGAN4 are similar to the original reported analysis [15], confirming the dominance of the ‘Firmicutes’ and ‘Bacteroidetes’ groups. The result is shown in Figure 4.

### Sequence comparison against NCBI-NR database

**Figure 5:**
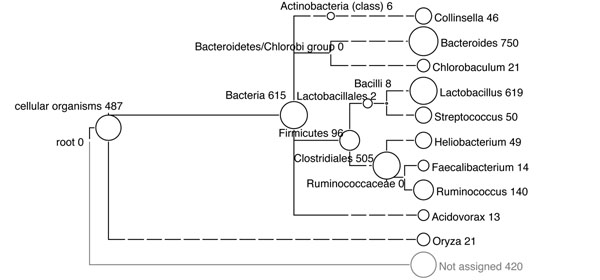
MEGAN 4 analysis result for ‘mice-data’ against the NCBI-NR database. Although we expected to obtain no significant hits, a wide range of hits were found. Numbers in taxonomic tree indicate the number of assigned reads to each taxon collapsed at ‘genus’ level of NCBI taxonomy.

The results obtained by comparing ‘mice-data’ against the NCBI-NR database are quite surprising (Figure 5). we expected to observe no hits, since the NCBI-NR database does not contain rRNA sequences. Any hits found should only appear by chance. However, the probability of observing random hits with an alignment score above the Min Score of 120 is quite low.

What we in fact observed is that a hit is found for most reads, usually to protein entry labeled “hypothetical protein”. Only a small number of reads (420) were not assigned to a taxon, and this was usually because the ‘min score’ threshold was not reached. While in some cases the taxonomic assignment based on *NR* was the same as the one obtained using an appropriate method, in most cases the assignment to was to a taxon that is probably incorrect. One of the best examples of the wrong assignment using NCBI-NR is the node *Oryza* (21 reads mapped to an unknown protein [*Oryza sativa Japonica* Group]), all of which should be assigned to *Lachnospiraceae* in the phylum *Firmicutes*. Because many of matches are highly significant, this all indicates that the NCBI-NR database probably contains a number of 16S rRNA sequences that are falsely assumed to be protein-coding genes.

An important practical implication of this study is that one should remove all rRNA sequences from a random shotgun dataset before performing an NCBI-NR based analysis, as they will lead to false positive assignments.

### Comparison with other methods

**Figure 6:**
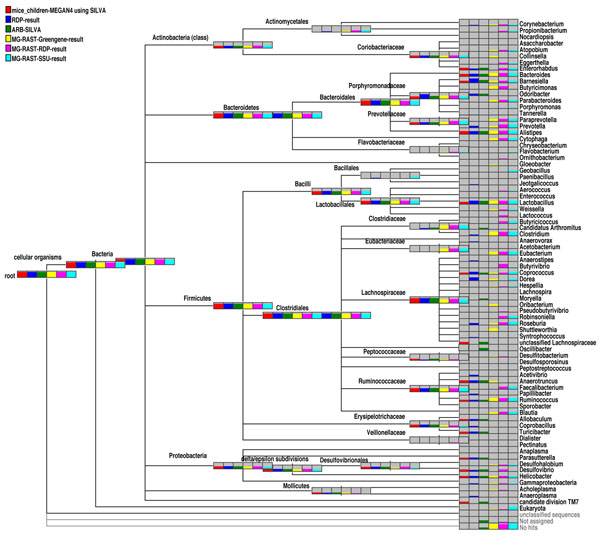
Comparison of the taxonomic analysis of a 16S rRNA dataset [15], computed using six different approaches: MEGAN4’s BLASTN-based SILVA analysis, the RDP website’s classifier [6], MG-RAST’s RDP-based approach [21], the SILVA website’s aligner [5], MG-RAST’s Greengene based approach and MG-RAST’s SILVA based approach targeting the SSU gene. In this figure, the bar charts on higher-rank nodes reflect the total number of reads assigned to the corresponding node or to any of the nodes in the subtree below the node.

In Figure 6 we show the comparison of the MEGAN 4’s SILVA-based analysis of 16S rRNA reads (depicted in Figure 4), with analyses produced using the other services mentioned above. All the nodes are scaled by the summarized value (sum of the reads to a particular node and the related children nodes). Only for MG-RAST results it was not possible to achieve the hierarchical assignments. Here we generally see a good correlation between all the analyses. In some cases where MEGAN can not attain a high number of hits at genus level (for example *Oscillibacter*), the reads are assigned to a higher level to meet the threshold.

## Conclusions

Metagenomics is a fast growing field and novel tools are required to analyze the ever growing datasets. Amplicon sequencing targeting the 16S rRNA gene is widely used for estimating the taxonomic structure of environmental bacterial assemblages. MEGAN 4, already widely used for analyzing random shotgun sequences, can now also be used for 16S rRNA, allowing the direct comparison of taxonomic profiles obtained from different types of data, and different methods.

## Competing interests

The authors declare that they have no competing interests.

## Author contributions

SM and DHH designed the project and wrote the manuscript. MS created the mapping file and wrote necessary codes. DHH wrote necessary codes for implementing this new approach in MEGAN. SM performed all the BLAST and comparisons.
